# Impact of Extrinsic Defects in Wavelength Separation Coatings on the Process of Laser-Induced Damage

**DOI:** 10.3390/mi16111191

**Published:** 2025-10-22

**Authors:** Shichen Shen, Xinda Zhou, Yinbo Zheng, Jie Li, Tianhao Zhang, Linjie Zhao, Liqun Chai, Mingjun Chen

**Affiliations:** 1 Research Center of Laser Fusion, China Academy of Engineering Physics, Mianyang 621900, China; ShiccShen@163.com (S.S.); zhouxinda2012@163.com (X.Z.); zybnb832@sina.com (Y.Z.); chailiqun@163.com (L.C.); 2 State Key Laboratory of Robotics and System, Harbin Institute of Technology, Harbin 150001, China; zhangthhit@163.com (T.Z.); zhaolinjie@hit.edu.cn (L.Z.); chenmj@hit.edu.cn (M.C.)

**Keywords:** wavelength separation coating, extrinsic defects, laser-induced damage process, multi-physics coupling model

## Abstract

Wavelength separation coatings can effectively separate the fundamental frequency (1ω) and third harmonic (3ω) laser beams. However, the laser-induced damage threshold (LIDT) of the surface defect-free WS coatings for the 3ω laser is 1.68 J/cm^2^ (obtained in the preliminary experiment), significantly lower than the ideal LIDT of the fused silica substrate (80 J/cm^2^). This is directly correlated with extrinsic defects such as nanoscale defects and nodular defects introduced during the coating manufacturing process. Moreover, the damage in WS coatings caused by extrinsic defects is a complex physical process involving multiple physical phenomena such as material melting, vaporization, and ejection. The mechanism by which extrinsic defects interact with lasers to form damage is not yet fully elucidated. To address this, a multi-physics coupling model considering photoelectric, thermal and stress was established to simulate the incident laser propagation within coatings, the temperature distribution and thermal stress distribution of the coating material. This model systematically investigates the influence of defect location, type, and size on the laser-induced damage process. It is found that when a 10 nm-diameter defect is located at the 32nd layer of the coatings, the light intensity enhancement factor (*LIEF*) for 3ω laser can reach up to 5 times that for the 1ω laser. The variation in thermal stress induced by changes in defect size is jointly determined by the defect-induced modulation effect and the interference effect realized by the coating. This work theoretically reveals the mechanism of extrinsic defects in the laser damage. It provides effective guidance for establishing control standards for extrinsic defects during the optical coating process.

## 1. Introduction

Wavelength separation coatings (WS coatings), serving as harmonic separation components, are widely employed in large-scale high-power laser systems due to their excellent spectral characteristics, ease of installation and adjustment, and space-saving advantages [1]. However, during the manufacturing process of optical coatings, whether through physical vapor deposition or chemical vapor deposition methods, extrinsic defects such as nanoscale absorption defects and nodular defects are inevitably introduced. These defects act as precursors for laser-induced coating damage, significantly reducing the laser-induced damage threshold (LIDT) and thereby limiting further enhancement of the output power and operational lifespan of laser systems [2,3,4,5,6]. Consequently, investigating the process of laser-induced damage in optical coatings initiated by extrinsic defects under nanosecond laser irradiation is of great significance.

The nanosecond laser-induced damage process caused by extrinsic defects introduced during manufacturing is often accompanied by various physical phenomena such as material melting, vaporization, and splashing [7,8]. Consequently, the initial energy deposition and subsequent evolution mechanisms of damage behavior induced by these defects have not been fully elucidated. Offline post-mortem characterization techniques such as Scanning Electron Microscopy (SEM), Focused Ion Beam (FIB), and Photoluminescence (PL) are employed to analyze and deduce the relevant damage mechanisms and processes [9,10,11]. Shan et al. adopted a three-dimensional spatiotemporally resolved method to observe the damage process during the interaction between optical coatings and lasers, obtaining accurate defect information of the films [12]. Liu et al. evaluated the laser-induced damage resistance of coatings using different LIDT testing protocols, revealing the influence of different polarizations on film failure by observing and comparing the damage characteristics of films under various protocols [13]. Gong et al. [14] experimentally investigated the origin and damage characteristics of nano-absorbing precursors under picosecond irradiation from aspects such as damage morphology, elemental composition, and splashing distribution. To further clarify the laser-induced damage growth process in coatings, Zhou et al. utilized time-resolved shadowgraphy to observe the dynamic evolution of damage, including ejection and delamination, in multilayer HfO_2_/SiO_2_ films under forward or reverse laser irradiation [15]. However, in the process of laser-induced damage, the influence of extraneous defects within coatings on the interaction between laser and materials cannot be directly obtained through experiments.

Meanwhile, to further explain the reason for the formation of damage pits induced by laser irradiation, theoretical derivation was chosen to reveal the mechanism of laser-induced coating damage. Stolz et al. investigated the influence of different oxide film materials, laser incidence angles, and film thicknesses on the incident laser intensity distribution through simulations [16], and also computed the effects of nodular defects generated by different coating processes on laser energy deposition [17]. Li et al. [18] conducted modeling and simulations to study the impact of the size, position, and geometric constants of nodular seeds in coatings on their forward- and backward-scattering characteristics. However, previous research has primarily focused on coatings with a single optical function. For WS coatings which transmit 1ω laser radiation while reflect 3ω laser radiation, the theoretical study of external defects on laser damage is still insufficient. Guo et al. investigated the damage induced in composite materials by a combination of femtosecond laser and continuous-wave laser and found that the damage effects achieved with different laser combinations exceeded those caused by a single laser [19]. The influence mechanism of external defects on the single-wavelength laser damage of WS coatings is not yet fully understood. Furthermore, the damage caused by lasers of different wavelengths due to nodular defects and nanoscale absorptive defects has not been quantitatively characterized. Therefore, this paper focuses on WS coatings, investigating the influence of various types and forms of nanoscale absorption defects and nodule defects introduced during the manufacturing process on the laser damage at different wavelengths. It theoretically analyzes the effects of extrinsic defects on the damage location, temperature, and thermal stress. This study provides important theoretical guidance for reducing extrinsic defects introduced during the manufacturing of optical coatings and enhancing their laser-induced damage resistance. At the same time, this study lays a foundation for the subsequent better exploration of the laser interaction mechanism at different wavelengths.

## 2. Model Establishment and Experiment

### 2.1. Sample Preparation and Damage Threshold Test

This work employs plasma-assisted electron beam deposition to coat a wavelength separation coating onto a fused silica (Corning 7980) substrate. Taking the wavelength of 1064 nm as the standard, the coating structure is air/(0.42H0.92L)^16^0.42H1.69L/substrate, where H represents the high-refractive-index material HfO_2_ with a quarter-wave optical thickness, and L denotes the low-refractive-index material SiO_2_ with a quarter-wave optical thickness.

**Figure 1:**
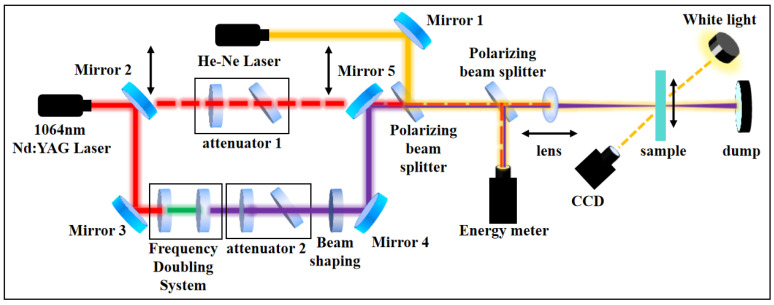
Schematic diagram of the optical path for the pulsed laser-induced damage test platform. By translating the lens, laser beams of different wavelengths are ensured to be focused onto the surface of the WS coatings device.

This study utilizes a laser-induced damage test platform to evaluate the laser damage resistance of wavelength separation coatings, as illustrated in Figure 1. A Nd:YAG nanosecond laser with a wavelength of 1064 nm is selected. Through a frequency doubling elements, the 1ω laser with a pulse width of 7 ns is converted into a 3ω laser with a wavelength of 355 nm and a pulse width of 5 ns. A He-Ne laser is employed for precise positioning of the test point, monitoring the status of components, and diagnosing the damage process, though it does not itself cause any damage. By synchronizing the movement of Mirror 2 and Mirror 5, switching between the infrared pulsed laser and the ultraviolet pulsed laser generated by the frequency-doubling system can be achieved. The energy density of the damaging laser is adjusted using a combination of a polarizer and a half-wave plate.

In the damage threshold test experiment, the R-on-1 method was adopted. This method involved continuously irradiating the same spot on the component surface with incrementally increasing laser flux at fixed time intervals until permanent characteristic changes occur in the optical coating surface. At each spot, each laser flux was applied only once, with a 4 s interval between exposures to eliminate the influence of heat accumulation on test results. Damage threshold tests were repeatedly conducted on 15 equally spaced measurement areas sequentially selected on the surface of the WS coatings. The laser flux on the coating sample was calculated based on the energy meter. The LIDT was measured to be 43.21 (±5.43) J/cm^2^ under a 7 ns pulse width 1ω irradiation, and 1.68 (±0.09) J/cm^2^ under a 5 ns pulse width 3ω laser irradiation.

**Figure 2:**
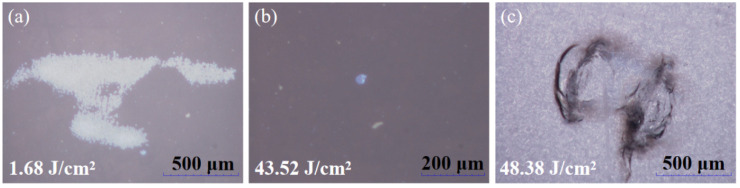
Characterization of damage morphology induced by laser irradiation at different wavelengths using optical microscopy. (**a**) Damage morphology generated by 3ω laser irradiation. (**b**,**c**) Damage morphology resulting from 1ω laser irradiation. The laser flux corresponding to each damage morphology is indicated in the lower left corner. The damage morphology induced by 3ω laser irradiation represents a mapping of the laser spot. In contrast, the damage morphology caused by 1ꞷ laser irradiation varies with laser flux, leading to either coating or substrate damage.

The damage morphologies induced by laser irradiation at different wavelengths under an optical microscope are shown in Figure 2. The damage pattern generated by 3ω laser irradiation corresponds to the mapping of the laser spot. In contrast, the LIDT of the 1ω laser exhibits a much wider variation range than that of the 3ω laser, and the damage morphologies generated at different test sites differ significantly, which may lead to coating or substrate damage. When the fluence of the 1ω laser significantly exceeds the LIDT, it simultaneously causes damage to the fused silica substrate and tearing of the coating. When the fluence of the 1ꞷ laser just reaches the LIDT, it leaves a single-point damage pit on the surface of the coating.

Finally, considering that the actual laser pulse width in the practical application of the optical component is 3 ns, and to facilitate the dissemination of the research results, the LIDT of the optical coating assembly (355 nm, 3 ns) was converted using the following equation [20]:

(1) $$LIDT(3\mathrm{ns})=LIDT\times {\left(\frac{3}{\tau }\right)}^{x}$$

where *LIDT* denotes the laser-induced damage threshold measured in the experiment. *τ* represents the pulse width of the laser during the experiment, with the fundamental frequency laser having a pulse width of 7 ns and the third-harmonic laser having a pulse width of 5 ns. The value of *x* is set to 0 when the incident laser is at the fundamental frequency, and to 0.35 when the incident laser is at the third-harmonic frequency. Therefore, the calculated LIDT (3 ns) under 1ꞷ laser irradiation is 32.12 J/cm^2^, while under 3ω laser irradiation, it is 1.68 J/cm^2^.

### 2.2. Establishment of the Multi-Physics Coupling Model

When laser beams of different wavelengths are incident on a WS coating, their phases and intensities are simultaneously modulated within the coating to form a standing wave, thereby achieving wavelength separation. Extrinsic defects introduced during the coating manufacturing process can interfere with the transmission of laser light in the electromagnetic field, altering the predetermined laser propagation path. This causes localized regions to form hot spots due to the superposition of laser intensity. Under subsequent high-power laser irradiation, free electrons around these hot spots in the coating are easily excited, leading to continuous ionization and the formation of plasma, resulting in energy deposition. These energy deposition points evolve into high-temperature and high-pressure regions under further laser exposure, ultimately causing damage to the coating.

**Figure 3:**
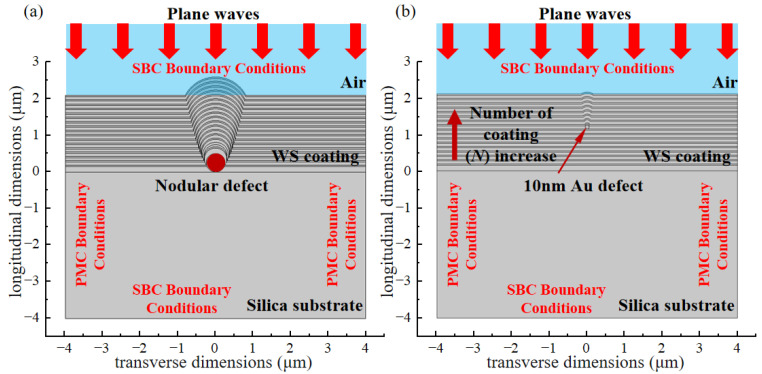
Modeling of extrinsic defect in optical coating. (**a**) Nodular defect; (**b**) 10 nm-diameter defect.

To investigate the influence of manufacturing-induced extrinsic defects on the interaction between laser and materials during laser-induced damage, Figure 3 presents an optical coating model incorporating extrinsic defects. In the manufacturing process of optical coatings, the actual structures of extrinsic defects vary. However, due to the influence of surface tension during the spraying process, common defects typically exhibit approximately circular or elliptical cross-sections [21]. Thus, the defects are simplified into circular shapes to improve computational efficiency and facilitate simulation results with better general applicability.

The dimensions of the simulation region are significantly smaller than those of actual coatings. Therefore, Perfect Magnetic Conductor (PMC) boundary conditions are applied on both sides of the model. The radiation and propagation of lasers in transparent optical materials can be considered as a plane electromagnetic wave incident from the surface of an optical component and propagating along the negative *y*-axis direction. Therefore, the scattering boundary condition (SBC) is set at the boundary corresponding to the incident direction of the plane electromagnetic wave. To guarantee the convergence of the simulation, the mesh size of the model must satisfy the Courant–Friedrichs–Lewy (CFL) condition [22]. With a wavelength of 355 nm as reference, λ/6 = 355 nm/6 = 59 nm. Thus, in this study, the model is discretized using small triangular elements with a side length of 55 nm.

Since the radiation and propagation of laser light in optical coatings can be regarded as the behavior of plane electromagnetic waves, the Finite Element Method (FEM) was applied to numerically solve Maxwell’s equations, which govern the propagation properties of electromagnetic waves. The light intensity *I* was used to characterize the concentration of energy as a laser beam passes through an optical coating [23].

(2) $$I=\left|\frac{1}{\tau }\int_{0}^{\tau }Sdt\right|=\frac{1}{2}\sqrt{\frac{\varepsilon_{0}\cdot \varepsilon_{ri}}{\mu_{0}\cdot \mu_{ri}}}{\left|E\right|}^{2}$$

In the equation, *S* represents the energy flux density, which denotes the energy vertically passing through a unit area per unit time. During experiments, it can be approximated using the average energy flux density <*S*> at the incident laser spot. *E* is the electric field strength, *ε*_0_ is the vacuum permittivity, *ε_ri_* is the relative permittivity of the coating material, *μ*_0_ is the vacuum permeability, and *μ_ri_* is the relative permeability of the coating material. The products *ε*_0_·*ε_ri_* and *μ*_0_·*μ_ri_* represent the absolute permittivity and absolute permeability of the coating material, respectively. The calculation formula for the <*S*> at the incident laser spot is as follows:

(3) $$<S>=\frac{U_{pulse}}{A\tau }$$

where *U*_pulse_ represents the single-pulse laser energy, *A* denotes the laser spot area, and *τ* is the pulse width. The optical field intensity is defined by the electric field strength. The <*S*> for the incident 1ω laser (FWHM = 3 ns) is 1.07 × 10^14^ W/m^2^, corresponding to an electric field intensity of 2.8 × 10^8^ V/m at the surface of the HfO_2_ coating material. The calculated <*S*> for the incident 3ꞷ laser (FWHM = 3 ns) is 5.60 × 10^12^ W/m^2^, which corresponds to an electric field intensity of 6.5 × 10^7^ V/m at the surface of the HfO_2_ coating material. To comparatively analyze the temperature rise induced by different defects under 1ω and 3ω laser irradiation, the incident electric field intensity was uniformly set to 1 × 10^8^ V/m.

The interaction between a laser and a coating material result in temperature variations both spatially and temporally within the material, requiring the solution of the heat conduction equation for accurate description [24].

(4) $$\rho_{i}{C_{P}}_{i}\frac{\partial T}{\partial t}-k_{i}\nabla^{2}T=Q$$

where *C_Pi_* is the isobaric heat capacity of the coating material, *ρ_i_* is the density of the coating material, *T* is the temperature within the coating material, *k_i_* is the thermal conductivity coefficient of the coating material, and *Q* represents the electromagnetic heat generation resulting from the interaction between the laser and the coating material. *Q* can be determined via the imaginary part of the material’s refractive index.

Under the irradiation of the incident laser, the plasma plume generated through ionization of the coating material continues to expand, resulting in a rapid increase in local temperature within the coating. This induces variations in the internal stress of the material. Once the thermal stress reaches the ultimate strength of the coating material, fracture occurs and causes damage. Therefore, the force balance equation is introduced for analytical purposes. Neglecting the internal stress produced during the deposition process, the thermal stress *σ_T_* can be expressed as [25]:

(5) $$\sigma_{T}=\frac{E_{i}}{1-\upsilon_{i}}\cdot \Delta \beta \cdot \Delta T$$

where *E_i_* is the Young’s modulus of the coating material. *υ_i_* is the Poisson’s ratio of the material. Δ*β* represents the difference in thermal expansion coefficients between adjacent coating layers, and Δ*T* is the temperature difference between neighboring layers. Neglecting the effect of defect size variation on material parameters. The simulation did not account for the deformation of the coating material under elevated temperatures. The specific simulation parameters of the coating materials are listed in Table 1.

## 3. Results and Discussion

Due to the size effect between nanosecond pulsed lasers and extrinsic defects, nanosecond lasers of different wavelengths are sensitive to extrinsic defects of varying sizes. 3ω lasers are more sensitive to nanoscale defects near the upper layer of coatings, primarily causing field-induced ionization damage. On the other hand, 1ꞷ lasers are more sensitive to submicron-scale defects at the film–substrate interface, mainly leading to thermomechanical damage induced by thermal stress [28,29].

### 3.1. Influence of Nanoscale Defect Location on the Modulation Effect of Laser

The size of nanoscale defects is very small, and they are typically located at the interfaces between coatings. To clarify the effects of nanoscale defect positions within WS coatings on the modulation of incident laser light fields at different wavelengths, the nanoscale defects are simplified as circular structures with a diameter of 10 nm made of Au material [21]. The light intensity enhancement factor (*LIEF*) is introduced, defined as

(6) $$LIEF=I_{E}/I_{P}$$

where *I_E_* represents the light intensity within the optical coating containing nanoscale defect, and *I_P_* represents the light intensity within the defect-free optical coating.

**Figure 4:**
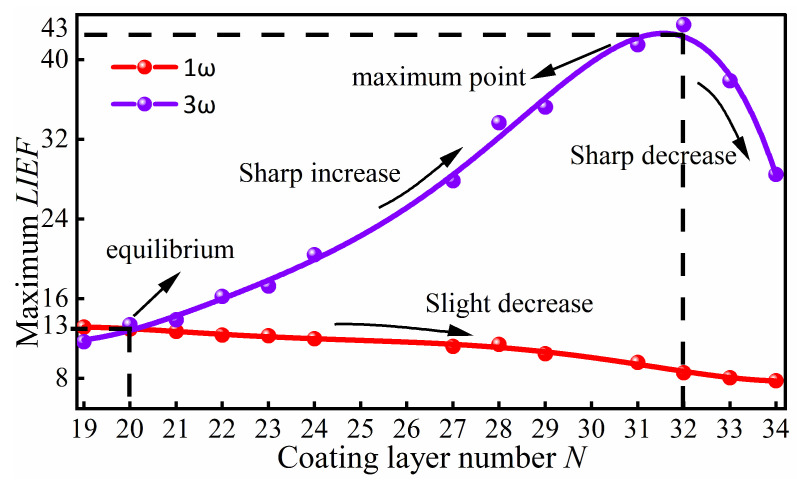
The relationship between the number of coating layers (*N*) containing a 10 nm-diameter defect and the maximum *LIEF* within the coating under lasers of different wavelengths. When *N* is 20, the *LIEF* generated by the defect is comparable for both the 1ω and 3ω lasers. When N is 32, the *LIEF* induced by the 3ω laser reaches its maximum, becoming 5 times that produced by the 1ω laser at the same location.

The influence of the layer number *N*, where a 10 nm-diameter defect is located, on the maximum *LIEF* within the optical coating for 1ω and 3ω lasers is shown in Figure 4. The results indicate that when a laser beam with a pulse width of 3 ns is incident perpendicularly, the *LIEF* induced by the 10 nm-diameter defect is essentially equal for both 1ω and 3ω lasers at *N* is 20. Subsequently, as *N* increases, the *LIEF* for the 3ω laser rapidly increases, while it decreases slowly for the 1ω laser. At *N* is 32, where the defect is only one periodic structure away from the surface, the *LIEF* for the 3ω laser reaches its maximum, which is 5 times that for the 1ω laser at the same location. However, when *N* exceeds 32, the periodic structure of the coating is disrupted, leading to a reduction in the *LIEF* for the 3ω laser as well.

**Figure 5:**
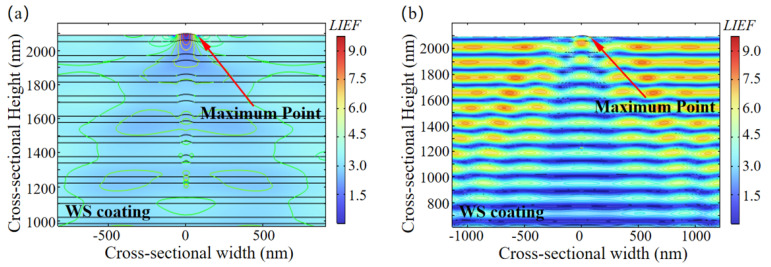
Distribution of *LIEF* in the coating under laser irradiation at different wavelengths when *N* is 20. (**a**) Irradiation laser wavelength of 1064 nm. (**b**) Irradiation laser wavelength of 355 nm. The field enhancement effect generated by 3ω laser irradiation is significantly superior to that of 1ω laser.

Figure 5 illustrates the distribution of the *LIEF* within the coating under irradiation by lasers of 1ω and 3ω when *N* is 20. As can be observed, the maximum *LIEF* for both the 1ω and 3ω lasers occurs at the interface between the nodular structure and the coating surface. When *N* is 20, although the maximum *LIEF* induced by a 10 nm-diameter defect is essentially identical for the 1ω and 3ω lasers, the field enhancement effect is more pronounced for the 3ω laser.

### 3.2. Influence of Nodule Defect Types on the Energy Deposition Process

Nodular defects are the most common extrinsic defects in optical coatings, which can be introduced during the coating manufacturing process through various means, such as the migration and accumulation of subsurface defects in fused silica substrates to the surface during heat treatment [29,30,31,32], impurities contained in the coating material during sputtering [33,34], or excess coating material. When a laser is incident on a WS coating, the presence of defects introduces an additional modulation effect on the laser. This causes more laser energy to accumulate at the defects near the coating–substrate interface, resulting in a temperature rise. The modulation effect of nodular defects on lasers of different wavelengths cannot accurately describe the interaction between the coating material and the laser.

**Figure 6:**
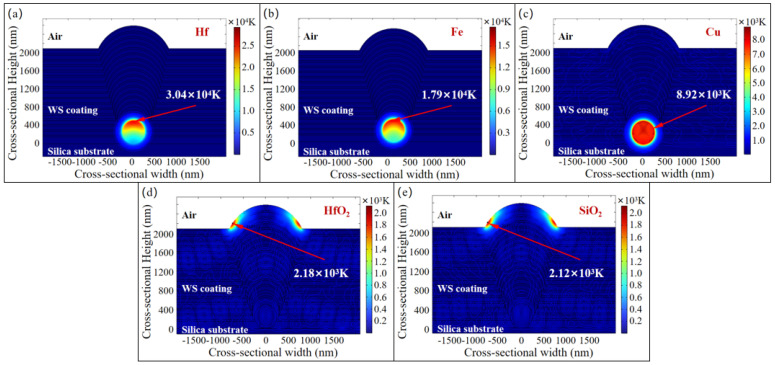
Simulation results of temperature distribution induced by different types of nodular defects under 3 ns pulse duration 1ω laser irradiation. (**a**) 500 nm Hf nodular defect; (**b**) 500 nm Fe nodular defect; (**c**) 500 nm Cu nodular defect; (**d**) 500 nm HfO_2_ nodular defect; (**e**) 500 nm SiO_2_ nodular defect.

**Figure 7:**
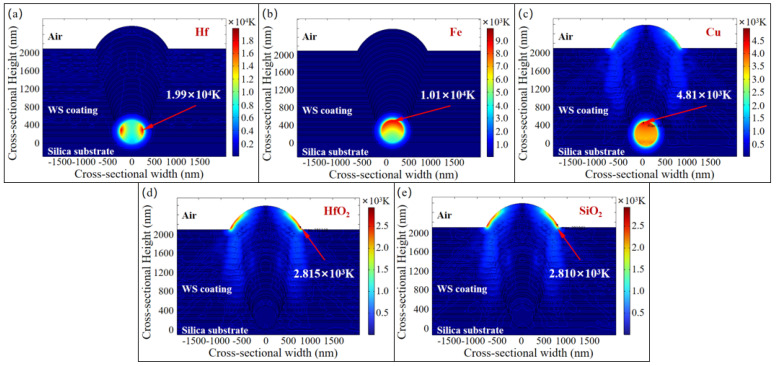
Simulation results of temperature distribution induced by different types of nodular defects under 3 ns pulse duration 3ω laser irradiation. (**a**) 500 nm Hf nodular defect; (**b**) 500 nm Fe nodular defect; (**c**) 500 nm Cu nodular defect; (**d**) 500 nm HfO_2_ nodular defect; (**e**) 500 nm SiO_2_ nodular defect.

Therefore, further consideration is given to the absorption of the laser energy by the defects. A model was constructed to calculate the temperature distribution and the location of the maximum temperature within the coating containing nodular defects after vertical irradiation by a 3 ns pulse laser at different wavelengths. Given that nodular defects can originate from various sources, the impact of different types of defects on the model was investigated. The metal defects, such as Hf, Fe, and Cu, and the coating material defects, including SiO_2_ and HfO_2_, were selected as research objects and simplified into circular shapes with a diameter of 500 nm. The temperature distribution results induced by different types of defects under 3 ns pulse lasers at 1ω and 3ω wavelengths are shown in Figure 6 and Figure 7.

The results demonstrate that for metallic defects, damage tends to originate from these impurities regardless of whether the vertically incident laser is at the 1ω or the 3ω. When the defect size and location are identical, the final temperature of Hf elements is higher than that of Cu and Fe elements. Both Fe and Hf elements can cause damage to the coating material (*T* > 10^4^ K, plasma formation). For coating material defects, the region with the highest temperature is located at the interface between the nodular structure and the top coating layer. When the defect size and location are the same, the peak temperature induced by SiO_2_ material shows comparatively negligible difference from that caused by HfO_2_ material. Furthermore, with the same incident laser and defect size, metallic defects reach higher temperatures compared to coating material impurities.

For metallic defects, the maximum temperature induced by 1ω laser exceeds that caused by 3ω laser for the same type of metallic defects. However, for coating material defects, the maximum temperature induced by 3ω laser surpasses that of 1ω laser. This phenomenon is likely directly related to the function of the WS coating, which is designed to transmit the 1ω laser while reflecting the 3ω laser via an interference effect. For metallic defects with high absorption coefficients, the temperature increase resulting from the redistribution of laser energy due to the modulation effect of nodular defects on the 3ω laser is insufficient to offset the temperature rise induced by the coating’s designed interference effect. On the other hand, for coating material defects with lower absorption coefficients, the modulation effect of nodular defects on the 3ω laser causes more laser energy to be concentrated around the defects, enhancing their absorption of laser energy and leading to a temperature increase that exceeds that caused by the interference effect. As the deformation of the coating material during the heating process was not considered, within the 3 ns simulation timeframe, the temperature rise is primarily concentrated around the metallic defects.

### 3.3. Influence of Nodular Defect Size on Thermal Stress Induced by Lasers

**Figure 8:**
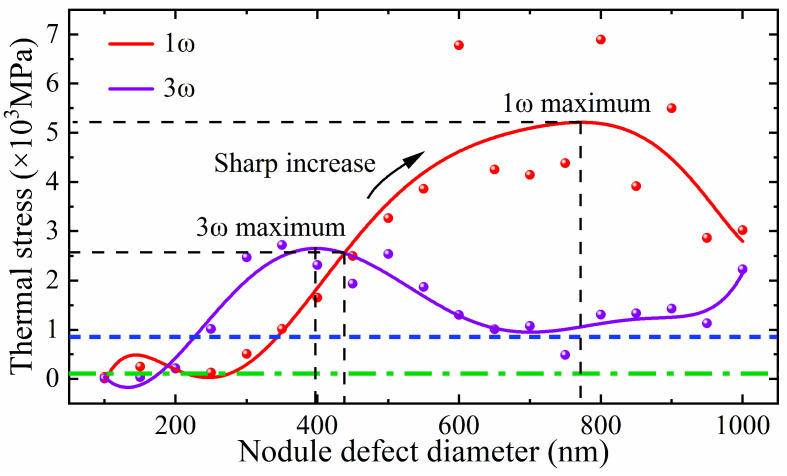
The relationship between nodule defect size and thermal stress under laser irradiation at different wavelengths. The blue dashed line represents the stress damage threshold of the coating material HfO_2_. The green dotted line indicates the stress damage threshold of the coating material SiO_2_. The thermal stress induced by the 1ꞷ laser increases rapidly within the range of nodular defect diameters from 250 nm to 770 nm.

The absorption of incident laser energy by defects leads to ionization and plasma generation, depositing laser energy within the coating. This not only causes a temperature rise but also induces thermal stress in adjacent coating materials due to temperature gradients. To further investigate the thermal stress distribution resulting from laser energy deposition, the force equilibrium equation is introduced to simulate and analyze the influence of nodular defect size variations on thermal stress under laser irradiation at different wavelengths. The laser beams, all vertically incident with a pulse width of 3 ns and a fluence of 1 × 10^8^ V/m, are applied. The nodular defects are composed of Au [21]. The relationship between nodular defect size and thermal stress induced by lasers of different wavelengths is illustrated in Figure 8.

From Figure 8, it can be observed, under identical laser flux irradiation, the thermal stress induced by 1ω laser irradiation experiences rapid growth within the defect diameter range of 245 nm to 772 nm, whereas the thermal stress generated by 3ω laser irradiation shows a similar rapid increase within the defect diameter range of 132 nm to 397 nm. This indicates that nodular defects exhibit greater sensitivity to thermal stress induced by 1ω laser irradiation. The maximum stress produced by the 1ω laser (with a defect diameter of 772 nm) reaches 5200 MPa, significantly exceeding the maximum stress of 2650 MPa generated by the 3ω laser (with a defect diameter of 397 nm).

When the defect diameters are 189 nm and 437 nm, the thermal stresses induced by 1ω and 3ω lasers are equal. Within the range of nodular defect size investigated, the thermal stress generated by the 3ω laser surpasses that of the 1ω laser only when the defect size falls between 189 nm and 437 nm. This indicates that within this specific diameter range, the thermal stress resulting from the modulation effect of the 3ω laser on nodular defects exceeds the combined thermal stress effects arising from both the modulation effect and the interference effect of the 1ω laser.

When the defect diameter is 175 nm and 230 nm, the thermal stress induced by the 3ω laser attains the stress damage thresholds of the coating materials SiO_2_ (110 MPa [35]) and HfO_2_ (850 MPa [36]), respectively. As the defect diameter continues to increase, the thermal stress reaches its maximum level under 3ω laser irradiation. However, once the defect size exceeds 397 nm, the lens-like focusing effect resulting from the modulation of the 3ω laser by the defect weakens, leading to a reduction in the laser energy deposited at the defect site and a consequent decrease in thermal stress. When the defect size surpasses 700 nm, the nodular structure grows synchronously with the defect size, allowing the 3ω laser to vertically pass through the nodular structure and irradiate the defect directly, resulting in an increase in thermal stress. At defect sizes of 275 nm and 345 nm, the thermal stress generated by the 1ω laser reaches the stress damage thresholds of SiO_2_ and HfO_2_, respectively. For defect sizes between 250 nm and 770 nm, the thermal stress induced by the 1ω laser increases rapidly. This is attributed to the combined effects of laser modulation by the defect and interference of the 1ω laser. However, when the defect size exceeds 770 nm, the lens-like focusing effect caused by the modulation of the 1ω laser diminishes, and the interference effect can no longer compensate for this reduction, leading to a decline in thermal stress.

**Figure 9:**
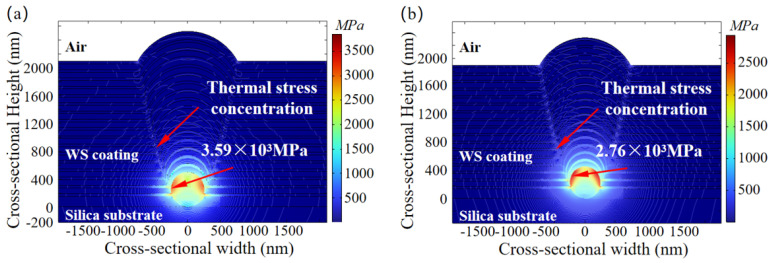
Simulation of thermal stress distribution induced by laser at different wavelengths on a 500 nm-diameter nodule defect. (**a**) Laser wavelength is 1064 nm. (**b**) Laser wavelength is 355 nm. The thermal stresses induced by 1ω and 3ω lasers at nodular defect sites are primarily concentrated near the metallic defect region, propagating simultaneously inward through both the substrate and the optical coating.

Calculation results of the thermal stress distribution induced by lasers of different wavelengths on a 500 nm-diameter nodular defect are shown in Figure 9. The results indicate that both vertically incident 1ω and 3ω laser-generated thermal stresses are primarily concentrated at the metallic defect interface, with the maximum stress point located at the junction between the base ideal coating layer and the defect. The thermal stress propagates simultaneously from the metallic defect toward both the substrate and the interior of the coating, with the most pronounced propagation direction being directly toward the optical coating surface. Stress concentration occurs at the transition region between the nodular defect structure and the ideal coating layer, causing the coatings inside the nodular structure to tear and delaminate during the damage process due to thermal stress effects.

**Figure 10:**
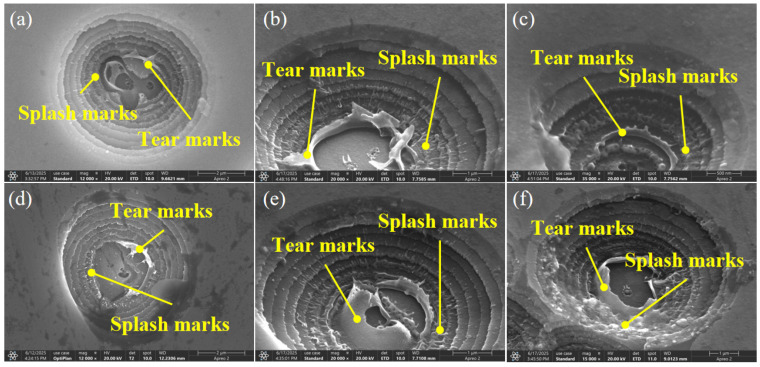
SEM morphology of damage crakes induced by 1ω and 3ω laser irradiation on surface defect-free wavelength separation coatings. (**a**–**c**) 1ω laser irradiation. (**d**–**f**) 3ω laser irradiation.

The microscopic morphology of isolated damage pits formed by laser irradiation on surface defect-free wavelength separation coatings is shown in Figure 10. The results indicate that the damage pits induced by 1ω laser irradiation are deeper than those generated by 3ω laser irradiation, with both exhibiting a stepped morphology. In practice, it is difficult to manufacturing coating components entirely free from extrinsic defects. Inevitably, nodular defects or nanoscale defects are introduced at the coating interfaces, which can subsequently lead to laser-induced damage. Based on experimental observations and the results of multi-physics coupling model simulations, it is demonstrated that 1ω laser radiation is more sensitive to nodular defects near the substrate, while 3ω laser radiation is more sensitive to nanoscale defects near the surface. Furthermore, distinct tear marks are observed at the bottom of the damage pits, along with evident ejection traces on the sidewalls. This indicates that as precursors to damage, a greater amount of laser energy is deposited in the vicinity of nodular defects and nanoscale defects, which is consistent with the energy deposition results predicted by the model. As laser energy accumulates around these defects, the thermal stress induced by temperature rise reaches the stress limit of the coating material, causing it to tear. When the temperature gradient within the coating propagates to the surface, stress concentration occurs at the interface between the nodular defect structure and the ideal coating layer under thermal stress, leading to coating delamination and the formation of step-shaped damage pits. However, the final morphology of the tearing of the coating at the bottom of the ablation crater was not observed in the simulation results, which may be attributed to the fact that the thermal explosion of the plasma plume was not considered in the simulation process.

## 4. Conclusions

Nanoscale absorbing defects and nodular defects within wavelength separation (WS) coatings act as damage precursors, serving as the primary cause for the reduction in the laser-induced damage threshold (LIDT). To address these issues, this paper establishes a multi-physics coupling model incorporating optical, thermal, and stress fields, taking into account the effects of light field propagation, electromagnetic field distribution, and thermal stress distribution during the damage process. The model is validated using actual damage crater morphologies. Furthermore, the influence of laser wavelength on the position, type, and size of defects during the damage process of WS coatings is investigated. Under same laser flux irradiation with fundamental frequency (1ω) and third harmonic (3ω) laser, the following conclusions can be drawn:

When a 10 nm-diameter defect is located within one periodic structure from the coating surface, the maximum laser-induced electric field (*LIEF*) value under 3ω laser irradiation is five times that under 1ω laser irradiation at the same position.

In the submicron size range, the maximum thermal stress induced by 1ω laser reaches twice that of the 3ω laser. The defect diameter range over which thermal stress continues to increase under 1ω laser irradiation is twice as large as that under 3ω laser.

For the same Hf, Fe, and Cu defects, the maximum temperature induced within the coating by 1ω laser exceeds that induced by 3ω laser. In contrast, for the same SiO_2_ and HfO_2_ defects, the maximum temperature induced by 3ω laser exceeds that induced by 1ω laser.

To sum up, this work systematically reveals the mechanism by which extrinsic defects induce damage under lasers of different wavelengths. The multi-physics coupling model established in this work is not only applicable to the interaction with WS coatings under nanosecond laser irradiation but can also be extended to other studies involving the same physical processes and mechanisms through the adjustment of laser or material parameters. Subsequent research will further consider changes in material properties during the laser damage process. This study provides effective guidance for establishing control standards for extrinsic defects in the optical coating process and is of significant importance for improving the laser resistance of coated components in optical systems.

## Figures and Tables

**Table 1:** Parameters of material employed in the calculation [26,27,28].

Parameter	Symbol/Unit	HfO_2_	SiO_2_	Au	Cu	Fe	Hf
Heat capacity	*C_P_*/(J/(Kg∙K))	270	750	129	380	440	140
Density	*ρ*/(Kg/m^3^)	9680	2203	19,300	8960	7870	13,310
Thermal conductivity coefficient	*k*/(W/(m·K))	2	1.38	10	350	60	20
Relative permittivity	*ε_r_*	3.9	2.25	−10.4	−10	−10	−5
Refractive index	*n*	1.97−0.000022*i*	1.475−0.00002*i*	0.18−3.1*i*	0.14−3.2*i*	2−3*i*	2.5−1.5*i*
Young’s modulus	*E*/(GPa)	170	190	79	120	211	
Poisson’s ratio	*υ*	0.27	0.17	0.42	0.34	0.29	
Coefficient of thermal expansion	*β*/(10^−6^ K^−1^)	5.6	0.5	14.2	16.5	11.8	

## Data Availability

The data that support the findings of this study are available from the corresponding author Jie Li upon reasonable request. The data are not publicly available due to institutional policies.
